# Correlation between oral microbial characteristics and overall bone density of Postmenopausal women based on macrogenomic analysis

**DOI:** 10.3389/fcimb.2025.1663645

**Published:** 2025-12-01

**Authors:** Man Liu, Min Wu, Yongjun Tang, Zhifeng Lin, Chanjuan Ye, Xixi Huang, Lu Zhou, Qiuying Lin, Dali Zheng, Youguang Lu

**Affiliations:** 1 Department of Preventive Dentistry, School and Hospital of Stomatology, Fujian Medical University, Fuzhou, China; 2 School of Medical Technology and Nursing, Shenzhen Polytechnic University, Shenzhen, China; 3 Stomatology Health Care Center, Shenzhen Maternity and Child Healthcare Hospital, Southern Medical University, Shenzhen, China; 4 Shenzhen Key Laboratory of Fermentation, Purification and Analysis, Shenzhen Polytechnic University, Shenzhen, China

**Keywords:** postmenopausal osteoporosis, oral microbiota, metagenomic, random forest model, biomarker

## Abstract

**Background:**

Postmenopausal osteoporosis (PMO), a prevalent bone disease triggered by estrogen deficiency - induced bone mass reduction and deterioration of bone tissue microarchitecture, escalates the risk of fragility fractures. Recent research has highlighted the pivotal role of oral and gut microbiota in PMO development, giving rise to the “oral - gut - bone axis” concept.

**Methods:**

A total of 21 postmenopausal women, aged 50 - 60, were recruited for the study. Based on bone mineral density (BMD) measurements from dual - energy X - ray absorptiometry (DXA), participants were divided into osteopenia, osteoporosis, and healthy groups. Saliva and dental plaque samples were collected for metagenomic sequencing to analyze microbial diversity and community composition, with differences identified via LEfSe analysis. KEGG pathway analysis was used to reveal variations in microbial functions. Based on these analyses, predictive models for bone density status were constructed using LASSO regression and random forest algorithms.

**Results:**

Significant differences in salivary microbial community structures were found between the osteoporosis and healthy groups (P = 0.041). LEfSe analysis revealed higher abundance of Aggregatibacter, Haemophilus haemolyticus, Haemophilus sputorum, Pasteurellaceae, Neisseria elongata, Aggregatibacter segnis, and Aggregatibacter aphrophilus in the osteopenia group, and higher abundance of Streptococcus pneumoniae and Haemophilus paraphrohaemolyticus in the osteoporosis group compared to the healthy group. The random forest models for osteopenia vs. healthy and osteoporosis vs. healthy yielded AUC values of 0.82 and 0.74, respectively, suggesting potential predictive capability, though further validation in larger cohorts is needed to confirm their generalizability. Functional analysis using LEfSe identified differential KEGG pathways, including glycan biosynthesis and metabolism in cancer, choline metabolism in cancer, and the cGMP-PKG signaling pathway.

**Conclusion:**

This exploratory study utilized metagenomic sequencing to analyze the relationship between oral microbiota and PMO while controlling for key confounders. We identified significant compositional and functional alterations in the oral microbiome associated with bone mineral density status, including specific bacterial species showing marked intergroup differences. A model based on differential microbial features exhibited preliminary discriminative capacity, and functional analysis suggested involvement of inflammatory and metabolic pathways. These findings provide initial evidence linking oral microbiota to PMO.

## Introduction

PMO is a condition that may increase the incidence of fragility fractures due to a decrease in bone mass and deterioration of bone tissue microarchitecture as a result of estrogen deficiency (Ma et al., 2024). PMO can lead to hip fracture, which may result in subsequent pain, deformity, functional impairment, and even mortality (AlYami et al., 2022). It is estimated that approximately 50% of adults aged 50 years and older suffer from osteoporosis (OP), of which approximately 70% are women with PMO (Yong and Logan, 2021). Due to the high prevalence and disabling nature of PMO, it has gained widespread attention and is emerging as a significant global health concern (Yong and Logan, 2021).

Previous studies have confirmed that gut microbiota influence bone homeostasis by altering host metabolism, the immune system, hormone secretion, and the gut-brain axis. Among these, short-chain fatty acids (SCFAs) play a major role in the gut-bone axis. The oral cavity and the gut harbour some of the most dense and diverse microbial communities. Although these two sites are physiologically distinct, they are directly connected and can influence each other in several ways (Kunath et al., 2024; Indrio and Salatto, 2025). The human oral cavity, colonized by over 700 microbial species, harbors one of the most complex and significant microbiomes in the body (Xian et al., 2018). Pathogenic oral bacteria, like Porphyromonas gingivalis, produce virulence factors such as lipopolysaccharides that activate the host immune system. They trigger the release of pro-inflammatory cytokines (IL-1β, IL-6, TNF-α) from macrophages and other immune cells, which in turn excessively stimulate osteoclast differentiation and suppress osteoblast activity (Singh et al., 2023). Moreover, oral microbial toxins, including gingipains, activate RANKL, accelerating osteoclast maturation and disrupting bone homeostasis (Cheng et al., 2021). Similarly, metabolic products from intestinal microbiota can permeate the compromised intestinal barrier and enter the systemic circulation. This triggers immune responses mediated by CD4+ T cells, leading to the production of factors that regulate osteoclast activity, ultimately activating osteoclasts and increasing bone resorption (Tu et al., 2020). Additionally, intestinal dysbiosis can cause an increase in pro - inflammatory cytokines like TNF - α and a decrease in anti - inflammatory cytokines such as IL - 10, and this inflammatory state can further worsen osteoporosis (Yang et al., 2022).

Building on evidence of individual roles for oral and gut microbiomes, the concept of the “oral-gut-bone axis” has emerged to encapsulate the bidirectional interplay among these sites and the skeleton (Tortora et al., 2023; Yamazaki, 2023). Oral microbiota can be transferred to the intestine through swallowing (Yamazaki and Kamada, 2024), where they disrupt the balance of gut microbiota and generate specific metabolic products. These metabolic products then activate immune and inflammatory responses and influence the balance of osteoblasts and osteoclasts through certain signaling pathways, such as those involving short-chain fatty acids and 5-hydroxytryptamine (Srivastava and Sapra, 2022; Zhao et al., 2022). These complex interactions highlight the significant role of the oral-gut-bone axis in the occurrence and progression of PMO.

However, despite the conceptual framework of the “oral-gut-bone axis,” the compositional and functional characteristics of the oral microbiota across different stages of bone loss, as well as its potential as a diagnostic tool, have not been fully explored. To address these gaps and clarify the role of the oral microbiota in PMO within the context of the “oral-gut-bone axis,” this study employed metagenomic sequencing to systematically analyze the oral microbiota of postmenopausal women stratified by BMD score (Osteopenia, Osteoporosis, Health). We aimed to identify oral microbial taxa, functional pathways, and metabolic potentials associated with the severity of PMO and to investigate their utility as non-invasive diagnostic biomarkers. Our findings are expected to offer novel insights into how the oral microbiota influences PMO through the oral-gut-bone axis, potentially providing new avenues for early detection or targeted interventions.

## Materials and methods

### Study subjects

The study subjects were recruited from the Oral Disease Prevention and Control Center of Shenzhen Maternal and Child Health Hospital. The inclusion criteria for the study subjects were as follows: (1) menopause for more than 1 year; (2) age between 50 and 60 years; (3) availability of a systemic bone mineral density test report and other related test reports following a general physical examination; (4) good health, with a body mass index (BMI) between 18.5 and 25, and no systemic diseases (the physical examination report showed no obvious abnormalities in any index); (5) periodontal health (BOP loci <10% for those with intact periodontal tissues and those with reduced periodontal tissues, and PD ≤3 mm), with more than 20 teeth preserved.

The exclusion criteria were as follows: (1) a history of smoking or alcohol abuse in the past three years (more than five times per week, with an average intake of more than 100 milliliters of white wine, half a catty of yellow wine, or five bottles of beer per occasion); (2) use of antibiotics, probiotics, or anti-osteoporosis medications in the past three months, such as sodium phenobarbital, carbamazepine, bisphosphonates, calcium supplements, or estrogen; (3) symptoms of gastrointestinal diarrhea or constipation in the past two weeks; (4) history of endocrine diseases, liver or kidney dysfunction, autoimmune diseases, or other related systemic diseases; (5) inability to understand the experimental protocol or cooperate with the clinical examination and procedures.

A total of 21 postmenopausal women were enrolled and underwent DXA from July 2024 to February 2025. Based on the results of DXA measurements, the study participants were divided into three groups: the osteopenia group (n = 7), the normal bone mineral density group (n = 7), and the osteoporosis group (n = 7). The following assessments were conducted for each participant: a basic information questionnaire, an oral health and periodontal examination, and metagenomic analysis of oral samples.

### Collection and testing of saliva and plaque samples

#### Saliva sample collection

The standard for saliva sample collection is based on the relevant criteria from Lu et al.'s study on the sampling strategy of the oral microbiome (Lu et al., 2022). Participants were instructed to refrain from brushing their teeth on the morning of the sampling day and the night before, and to avoid eating for at least 2 hours prior to sampling. Non-stimulated saliva was collected directly using a saliva collector. The collected volume was approximately 5 ml. Each sample was sealed, labeled with a unique sample number, and immediately stored in dry ice. The samples were then transferred to a -80°C freezer within 2 hours of collection.

#### Oral supragingival plaque collection

Supragingival plaque was collected from teeth with visible plaque at the gingival margin for each study subject. The selected teeth were isolated from the rest of the mouth using a sterile cotton swab. A sterile periodontal curette was then used to scrape the plaque from the gingival margin towards the coronal side. The collected plaque was placed into a sterile 1.5 ml centrifuge tube, and 1 ml of sterile 0.1 M phosphate buffer solution (PBS, pH 7.0) was added. The sample was then stored at -80°C. The standard for collecting oral supragingival plaque samples is derived from the criteria outlined in Lu et al.'s research on the sampling strategy of the oral microbiome (Lu et al., 2022).

#### Sample DNA extraction and quality control

##### DNA extraction and purification procedure

###### Thawing and initial centrifugation

Thaw the samples at room temperature. Vortex for 30 seconds to fully disperse the samples, followed by centrifugation at 2600×g for 10 minutes to precipitate food residues and epithelial cells.

###### Supernatant processing

Transfer the supernatant to a new tube and centrifuge at 13,000×g at 4°C for 10 minutes. Discard the supernatant and retain the bacterial pellet for DNA extraction.

###### Genomic DNA extraction

Extract genomic DNA using the QIAamp DNA Mini Kit (Qiagen, Hilden, Germany) according to the manufacturer’s standard protocol.

###### Microbiome DNA enrichment

Using the NEB Next Microbiome DNA Enrichment Kit (New England Biolabs, Ipswich, MA, USA), incubate 4 µg of DNA with 160 µL of MBD-Fc-bound glass beads at room temperature for 1 hour to separate host DNA from microbial DNA, thereby reducing human DNA contamination and enriching microbial DNA.

###### Ethanol precipitation

Purify the enriched microbial DNA using ethanol precipitation.

###### DNA quality assessment

Measure the DNA concentration and purity (A260/A280 ratio) using a Nanodrop™ 2000 spectrophotometer (Thermo Fisher Scientific, Wilmington, USA).

Agarose Gel Electrophoresis: Assess nucleic acid integrity and fragment size by 1% agarose gel electrophoresis.

Storage: Store the extracted DNA at -80 °C for subsequent use.

### Library construction and sequencing

Library Construction: Qualified DNA samples were fragmented into small segments of approximately 350 bp using an ultrasonic pulverizer. The DNA fragments were then processed using the Illumina Paired-End Library Preparation Kit. This involved repairing and blunting the DNA ends, followed by the addition of an A-tail at the 3’ end. Subsequently, distinct sequencing adapters were ligated to both ends of the DNA fragments. The DNA fragments with adapters were denatured from double-stranded to single-stranded to construct a single-stranded DNA library. Using the Illumina cBot Cluster Generation System, the DNA fragments underwent approximately 28 cycles of bridge amplification and denaturation. This process concentrated the fragments into clusters at their respective locations, achieving the signal intensity required for sequencing. This resulted in the formation of linearized monoclonal DNA cluster sequencing units suitable for the subsequent synthetic sequencing process.

Sequencing: The sequencing reaction was initiated on the Illumina PE150 sequencer by adding primers, DNA polymerase, and four types of dNTPs labeled with distinct fluorescent tags into the reaction system. The sequencer operated in the paired-end (PE) sequencing mode. The optical system within the sequencer detected and quantified the fluorescent signals emitted during each cycle, which were then used to calculate the sequence information of the DNA fragments.

### Sequencing data processing

Data Quality Control: High-quality reads were retained by sequentially removing reads containing more than 10 N bases, reads with a high proportion of low-quality bases, and reads likely originating from the host. Only reads with an average quality score above 20 and a length exceeding 50 bp were kept for further analysis.

Gene Assembly: Clean data obtained from quality control were assembled using the SOAPdenovo assembly software. After single-sample assembly, unused reads were pooled for hybrid assembly. The resulting scaffolds were broken at N junctions to obtain N-free sequence fragments, termed “scaftigs.” Scaftigs smaller than 500 bp were filtered out to prepare for subsequent gene prediction analysis.

Gene Prediction: Open reading frame (ORF) prediction was performed on the single-sample and hybrid-assembled scaftigs using MetaGeneMark. Redundant sequences were removed using CD-HIT software to construct a non-redundant gene set, referred to as the gene catalogue (Unigenes), for downstream bioinformatics analysis.

Raw sequencing data were imported into QIIME2 (v.2022.08) software for merging paired-end sequences. The DADA2 algorithm was used for noise reduction and extraction of amplicon sequence variants (ASVs). Sequences with an average quality score below 20 were filtered out, and samples with fewer than 4,000 ASVs were removed. ASVs with sequencing counts greater than 10 and detected in at least 18 specimens were retained to minimize the impact of low-abundance bacterial species on the false discovery rate (FDR). The Naive Bayes classifier was applied to match the Greengenes 2022 database for species annotation of representative amplicons, yielding the composition of all samples and calculating the relative abundance of bacteria at the species level.

### Statistical analysis

Quantitative data, after normality testing, were described using means ± standard deviations. Categorical variables were described via case counts and composition ratios. Fisher’s exact probability test and analysis of variance were employed to compare basic characteristics and epidemiological factors among the Osteopenia, Osteoporosis, and Health groups. To validate the sequencing stability of our study samples, we calculated dilution curves for observed species/ASVs based on sequencing depth for each sample. To reduce sampling variability, we averaged results from repeated resampling and plotted confidence intervals. We generated cumulative species boxplots using random resampling (multiple iterations) to visualize the distribution of newly added species across sample sizes. Good’s coverage was calculated and summarized; coverage values for each sample are provided in the **Supplementary Table**. Alpha diversity among groups was assessed using the Shannon index and Chao 1 index, with nonparametric tests employed to determine differences. β-diversity analysis employed principal coordinate analysis (PCoA) based on Bray-Curtis and Jaccard distances. Simultaneously, the vegan::adonis2 module conducted distance-based multivariate analysis of variance (PERMANOVA) with the model distance ~ group + BMI + year. Marginal effects were reported using by=“margin” to account for covariates, with 9,999 permutations. Simultaneously, betadisper and permutest were employed to assess homogeneity of dispersion within groups, preventing misinterpretation of “dispersion variation” as “center variation.” To visualize separation trends after covariate control, partial dbRDA (Condition(BMI + year)) based on capscaling was further conducted, with significance evaluated via permutation tests.

The LEfSe (without FDR correction) method identified differentially abundant microbial communities between the osteoporosis group and healthy controls, as well as between the osteopenia group and healthy controls, using an LDA score threshold of 2. LEfSe method identified differentially abundant microbial communities between the osteoporosis group and healthy controls, as well as between the osteopenia group and healthy controls, using an LDA score threshold of 2. LASSO regression further refined feature microbe selection from LEfSe results. Based on these features, random forest models were developed to evaluate classification performance in distinguishing Osteopenia vs. Health and Osteoporosis vs. Health samples. Model performance was assessed using ROC-AUC, and calibration and decision curves were plotted to evaluate predictive accuracy and clinical utility. LEfSe was also used to identify significantly different KEGG pathways across the three groups. All analyses were two-tailed with α = 0.05 and conducted in R (v4.3.2).

## Results

### Baseline characteristics of study participants

A total of 21 participants were enrolled and categorized into three groups based on BMD measurements obtained via DXA, in accordance with WHO diagnostic criteria: the Osteopenia group (n = 7), the Osteoporosis group (n = 7), and the Health group (n = 7). Comparative analysis of demographic and clinical characteristics showed no statistically significant differences in baseline parameters among the three groups (**Tables 1**–**3**).

**Table 1:** Comparison of baseline characteristics among study groups.

Variables	Overall (n=21)	Osteopenia (n=7)	Osteoporosis (n=7)	Health (n=7)	*P*
Ethnicity (%)					
Han	21 (100.0)	7 (100.0)	7 (100.0)	7 (100.0)	1
Occupation (%)					
Non-medical	17 (81.0)	6 (85.7)	6 (85.7)	5 (71.4)	0.734
Medical	4 (19.0)	1 (14.3)	1 (14.3)	2 (28.6)	
Education level (%)					
Associate/Bachelor	16 (76.2)	4 (57.1)	6 (85.7)	6 (85.7)	0.35
Master and above	5 (23.8)	3 (42.9)	1 (14.3)	1 (14.3)	
Household income (%)					
6000–20000 RMB	4 (19.0)	1 (14.3)	1 (14.3)	2 (28.6)	0.886
20000–50000 RMB	13 (61.9)	4 (57.1)	5 (71.4)	4 (57.1)	
Above 50000 RMB	4 (19.0)	2 (28.6)	1 (14.3)	1 (14.3)	
Smoking (%)					
Non-smoker	20 (95.2)	6 (85.7)	7 (100.0)	7 (100.0)	0.35
1–3 cigarettes/day	1 (4.8)	1 (14.3)	0 (0.0)	0 (0.0)	
Alcohol consumption (%)					
Never drink	10 (47.6)	3 (42.9)	3 (42.9)	4 (57.1)	0.826
Occasionally drink	11 (52.4)	4 (57.1)	4 (57.1)	3 (42.9)	
Dietary HABITS (%)					
Vegetarian	2 (9.5)	1 (14.3)	0 (0.0)	1 (14.3)	0.575
Balanced diet	19 (90.5)	6 (85.7)	7 (100.0)	6 (85.7)	
Exercise habits (%)					
Once a week	6 (28.6)	3 (42.9)	1 (14.3)	2 (28.6)	0.528
1–3 times a week	11 (52.4)	4 (57.1)	4 (57.1)	3 (42.9)	
Daily	4 (19.0)	0 (0.0)	2 (28.6)	2 (28.6)	
Chronic diseases (%)					
No	19 (90.5)	5 (71.4)	7 (100.0)	7 (100.0)	0.11
Yes	2 (9.5)	2 (28.6)	0 (0.0)	0 (0.0)	
Antibiotic use in the past 3 months (%)					
No	20 (95.2)	7 (100.0)	7 (100.0)	6 (85.7)	0.35
Yes	1 (4.8)	0 (0.0)	0 (0.0)	1 (14.3)	
Antibiotic use in the past 3 days (%)					
No	21 (100.0)	7 (100.0)	7 (100.0)	7 (100.0)	1
Dental cleaning in the past 6 months (%)					
No	21 (100.0)	7 (100.0)	7 (100.0)	7 (100.0)	1
Age	53.7 ± 2.2	52.9 ± 1.1	54.7 ± 3.1	53.6 ± 1.6	0.285
Weight (Kg)	66.5 ± 31.9	58.3 ± 5.7	56.3 ± 6.7	85.0 ± 52.2	0.174
Height (cm)	151.7 ± 30.3	164.6 ± 4.6	159.9 ± 6.3	130.7 ± 47.0	0.069
Years Since Menopause	1.6 ± 0.9	1.1 ± 0.4	2.0 ± 0.8	1.6 ± 1.1	0.187

**Table 2:** Comparison of oral health behaviors among study groups.

Variables	Overall (n=21)	Osteopenia (n=7)	Osteoporosis (n=7)	Health (n=7)	*P*
Daily toothbrushing frequency (%)					
1–2 times	18 (85.7)	6 (85.7)	6 (85.7)	6 (85.7)	1.000
3 times	3 (14.3)	1 (14.3)	1 (14.3)	1 (14.3)	
Brushing method (%)					
Horizontal	2 (9.5)	0 (0.0)	1 (14.3)	1 (14.3)	0.886
Vertical	13 (61.9)	5 (71.4)	4 (57.1)	4 (57.1)	
Mixed	6 (28.6)	2 (28.6)	2 (28.6)	2 (28.6)	
Post-meal oral hygiene (%)					
No	3 (14.3)	2 (28.6)	0 (0.0)	1 (14.3)	0.311
Yes	18 (85.7)	5 (71.4)	7 (100.0)	6 (85.7)	
Regular toothbrush replacement (%)					
No	2 (9.5)	1 (14.3)	1 (14.3)	0 (0.0)	0.575
Yes	19 (90.5)	6 (85.7)	6 (85.7)	7 (100.0)	
Regular dental check-ups needed (%)					
No	1 (4.8)	0 (0.0)	1 (14.3)	0 (0.0)	0.350
Yes	20 (95.2)	7 (100.0)	6 (85.7)	7 (100.0)	
Gum bleeding treatment needed (%)					
Generally Not Needed	9 (42.9)	3 (42.9)	4 (57.1)	2 (28.6)	0.330
Severe Bleeding Needed	7 (33.3)	1 (14.3)	3 (42.9)	3 (42.9)	
Need Medical Examination and Treatment	5 (23.8)	3 (42.9)	0 (0.0)	2 (28.6)	
Dental restoration needed for damage or loss (%)					
Needs Restoration	21 (100.0)	7 (100.0)	7 (100.0)	7 (100.0)	1.000
Daily Brushing Time	1.0 ± 0.3	1.0 ± 0.6	1.0 ± 0.0	1.0 ± 0.0	1.000
Toothbrush Replacement Time	2.3 ± 0.6	2.6 ± 0.5	2.1 ± 0.7	2.3 ± 0.5	0.387
Regular Dental Check-up Time	2.7 (0.6)	2.7 (0.5)	2.6 (0.8)	2.9 (0.4)	0.658

**Table 3:** Comparison of oral periodontal examination conditions among study groups.​.

Variables	Overall (n=21)	Osteopenia (n=7)	Osteoporosis (n=7)	Health (n=7)	*P*
Plaque Index	1.8 ± 0.6	1.8 ± 0.4	1.7 ± 0.8	1.8 ± 0.5	0.978
BI Bleeding Index	2.1 ± 0.6	1.8 ± 0.5	2.2 ± 0.7	2.3 ± 0.4	0.246
Probing Depth (PD)	2.8 ± 0.4	2.7 ± 0.3	2.9 ± 0.4	2.9 ± 0.5	0.615
Attachment Loss (AI)	2.7 ± 0.4	2.6 ± 0.3	2.8 ± 0.4	2.7 ± 0.4	0.755

### Sequencing depth analysis

Species accumulation boxplot analysis indicates that the number of detected species increases with sample size and gradually stabilizes (**Supplementary Figure 1**). Dilution curve analysis shows that the curve stabilizes at sampling depths exceeding 4,000,000 (**Supplementary Figure 2, 3**). Goods coverage analysis demonstrates microbial coverage, where higher values correspond to lower probabilities of undetected novel species in the sample (**Supplementary Table 1**).

### Comparison of oral microbiome diversity

**Figure 1:**
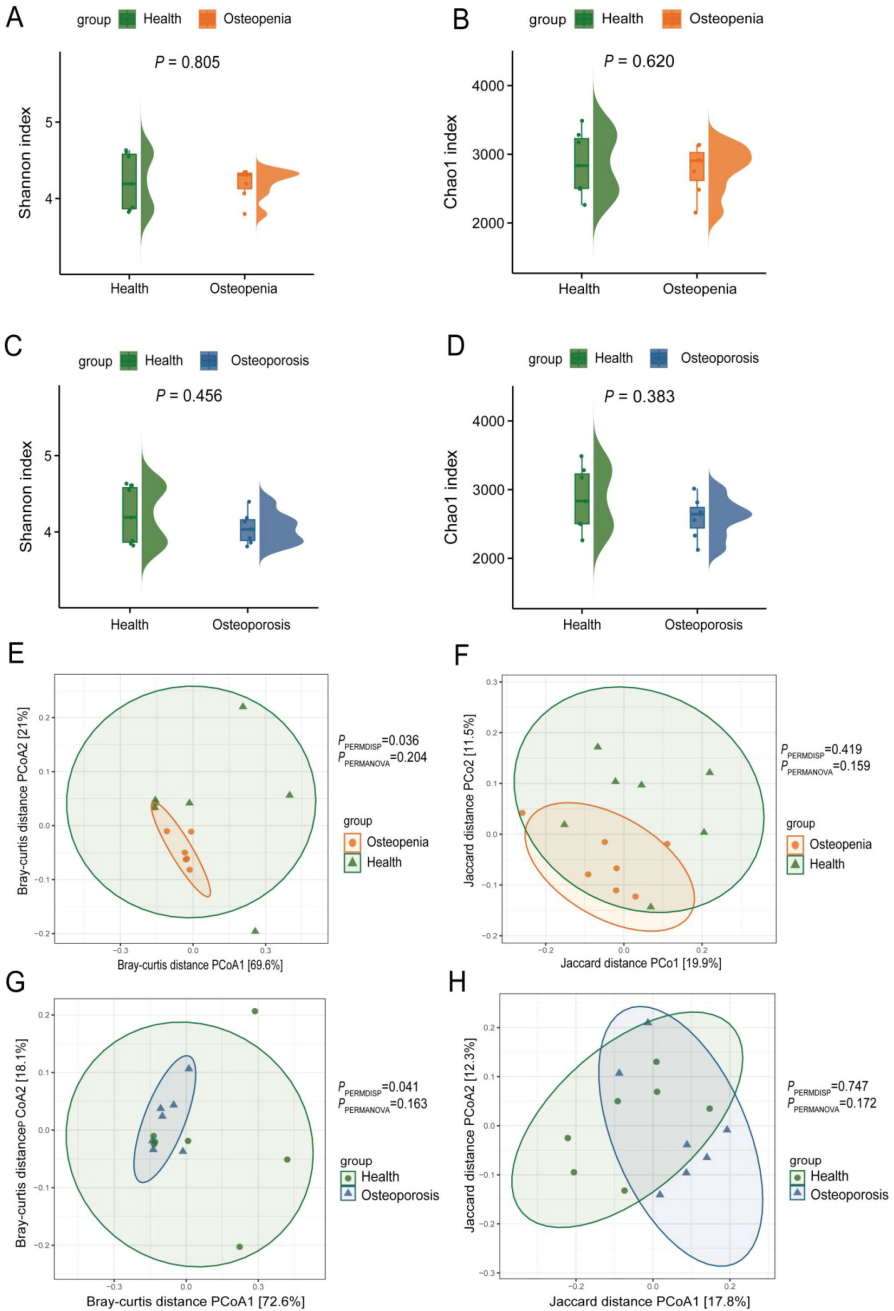
Comparison of salivary microbiota diversity. **(A)** Shannon Index for the Osteopenia group compared to the Health group. **(B)** Chao 1 Index for the Osteopenia group compared to the Health group. **(C)** Shannon Index for the Osteoporosis group compared to the Health group. **(D)** Chao 1 Index for the Osteoporosis group compared to the Health group. **(E)** PCoA analysis based on Bray-Curtis distance showing microbial beta diversity between the Osteopenia group and the Health group. **(F)** PCoA analysis based on Jaccard distance showing microbial beta diversity between the Osteopenia group and the Health group. **(G)** PCoA analysis based on Bray-Curtis distance showing microbial beta diversity between the Osteoporosis group and the Health group. **(H)** PCoA analysis based on Jaccard distance showing microbial beta diversity between the Osteoporosis group and the Health group.

**Figure 2:**
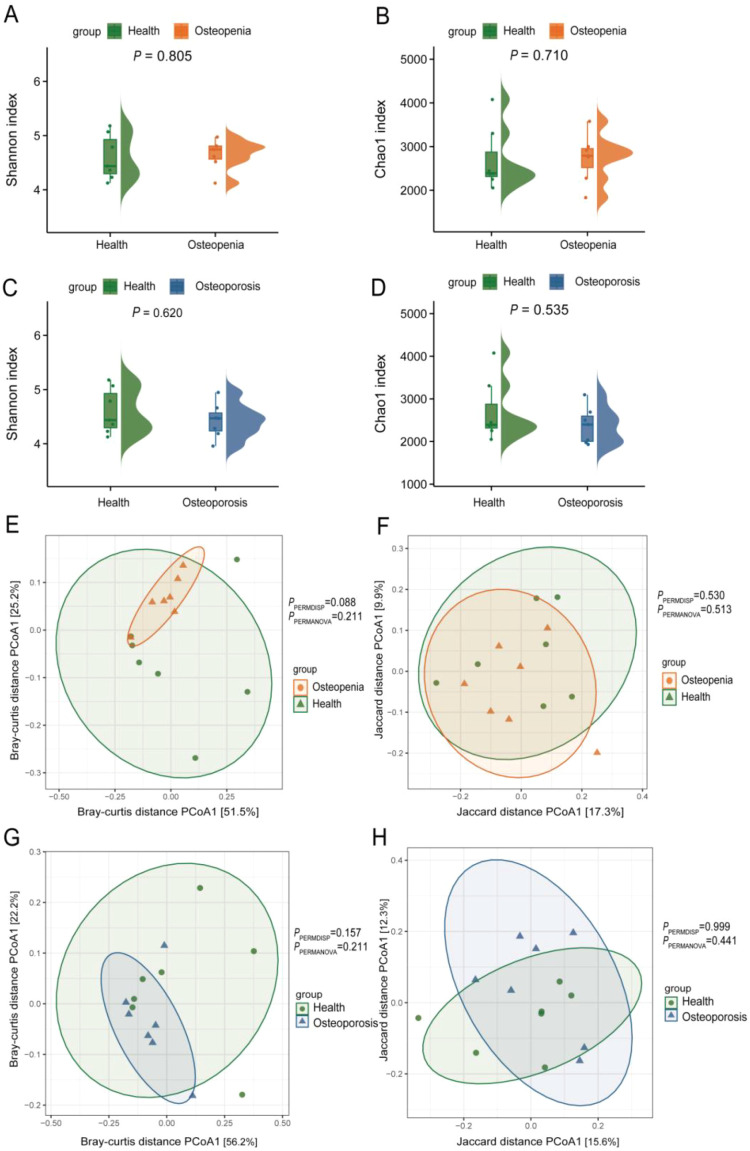
Comparison of dental plaque microbiota diversity. **(A)** Shannon Index for the Osteopenia group compared to the Health group. **(B)** Chao 1 Index for the Osteopenia group compared to the Health group. **(C)** Shannon Index for the Osteoporosis group compared to the Health group. **(D)** Chao 1 Index for the Osteoporosis group compared to the Health group. **(E)** PCoA analysis based on Bray-Curtis distance showing microbial beta diversity between the Osteopenia group and the Health group. **(F)** PCoA analysis based on Jaccard distance showing microbial beta diversity between the Osteopenia group and the Health group. **(G)** PCoA analysis based on Bray-Curtis distance showing microbial beta diversity between the Osteoporosis group and the Health group. **(H)** PCoA analysis based on Jaccard distance showing microbial beta diversity between the Osteoporosis group and the Health group.

This section compares the diversity of salivary and dental plaque microbiomes among subjects in the pre-osteoporosis, osteoporosis, and healthy control groups. Analysis of α diversity in salivary samples using Shannon and Chao 1 indices revealed no significant differences between the pre-osteoporosis and healthy groups, nor between the osteoporosis and healthy groups (**Figures 1A–D**). PCoA analysis based on Bray-Curtis distance revealed significant differences in salivary microbial community β-diversity among the pre-osteoporosis, osteoporosis, and healthy groups (PPERMDISP = 0.036, PPERMDISP = 0.041) (**Figures 1E, G**). In contrast, PCoA analysis based on Jaccard distance did not reveal significant differences among the three groups (PPERMDISP = 0.419, PPERMDISP = 0.747) (**Figures 1F, H**). After adjusting for BMI and year, the group effect of Health group vs Osteoporosis group became marginally significant under both Bray–Curtis and Jaccard measures (Bray: R²=0.201, F = 2.78, P = 0.091; Jaccard: R²=0.102, F = 1.35, P = 0.061), with neither covariate significant (BMI: P = 0.950; year: P = 0.226). BETADISPER indicated significant dispersion differences between groups (P = 0.030), suggesting variation may be partially influenced by dispersion; after controlling covariates, partial dbRDA showed overall P≈0.09 for group factors. In contrast, the group effect for Health group vs Osteoporosis group was not significant (Bray: R²=0.075, P = 0.345; Jaccard: R²=0.076, P = 0.459), but dispersion was significantly unequal (P = 0.021), with partial dbRDA showing no significance (P≈0.35) (**Supplementary Table 2**). In dental plaque samples, α-diversity analysis using Shannon and Chao 1 indices similarly revealed no significant differences between the pre-osteoporosis group and the healthy group, nor between the osteoporosis group and the healthy group (**Figures 2A–D**). PCoA analysis based on Bray-Curtis and Jaccard distances indicated no significant differences in plaque microbiota β-diversity between the pre-osteoporosis group and the healthy group, nor between the osteoporosis group and the healthy group (**Figures 2E–H**). After adjusting for BMI and year, group effects were not significant under either Bray–Curtis or Jaccard metrics for either Health group vs Osteopenia group or Health group vs Osteoporosis group comparisons (Health group vs Osteopenia group: Bray P = 0.176, Jaccard P = 0.257; Health group vs Osteoporosis group: Bray P = 0.407, Jaccard P = 0.837), and covariates were also insignificant. Intra-group homogeneity of dispersion tests were not significant for H vs P (P = 0.142) and marginally not significant for Health group vs Osteoporosis group (P = 0.090). Both overall and group-specific partial dbRDA analyses yielded non-significant results (Health group vs Osteopenia group: P = 0.180; Health group vs Osteoporosis group: P = 0.400) (**Supplementary Table 2**).

### Characteristics of oral microbiota composition

**Figure 3:**
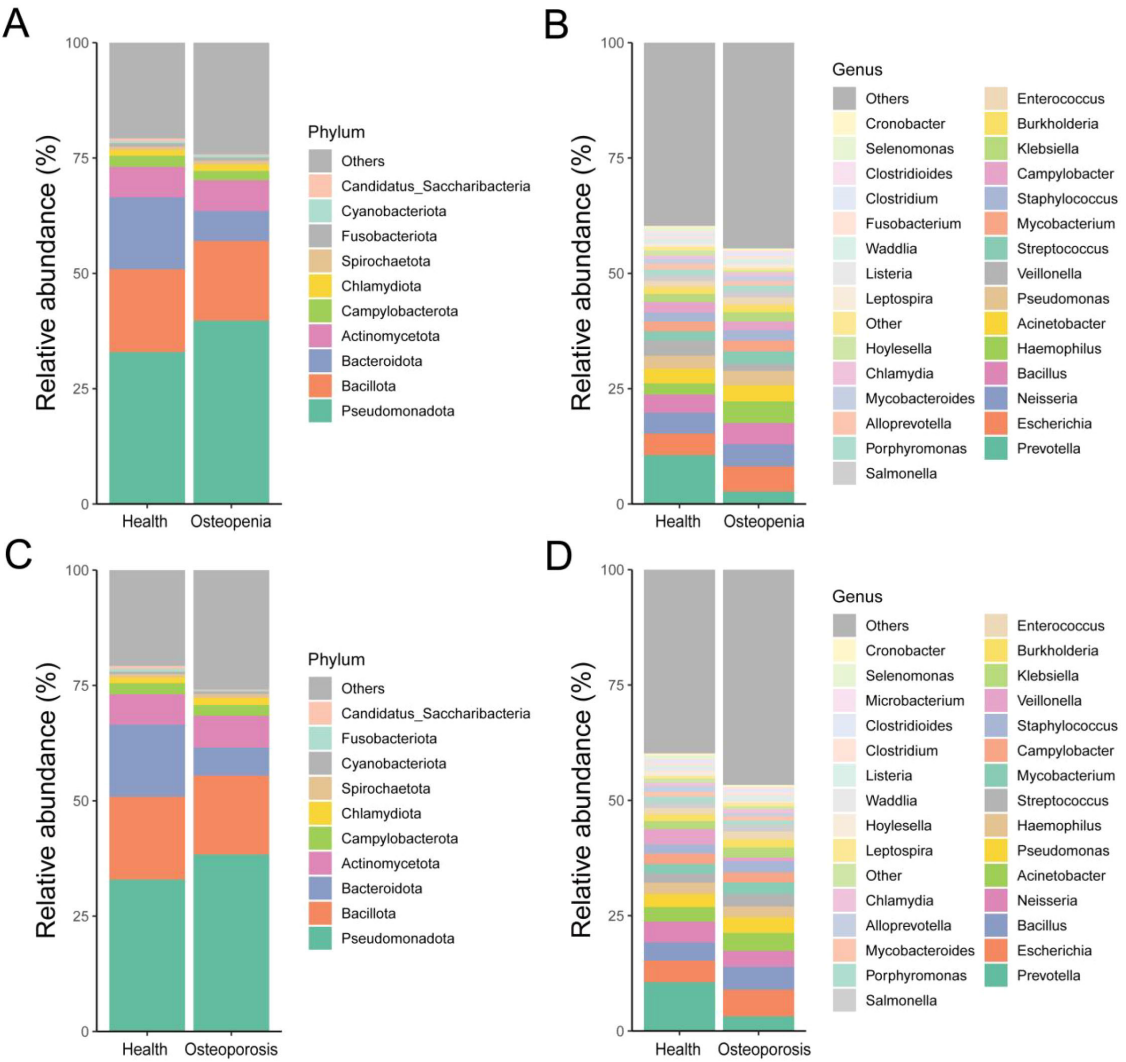
Distribution of salivary microbiota composition. **(A)** Distribution characteristics of microbial phyla between the Osteopenia group and the Health group. **(B)** Distribution characteristics of microbial genera between the Osteopenia group and the Health group. **(C)** Distribution characteristics of microbial phyla between the Osteoporosis group and the Health group. **(D)** Distribution characteristics of microbial genera between the Osteoporosis group and the Health group.

In saliva samples, the dominant phyla shared among the Health, Osteopenia, and Osteoporosis groups were Campylobacterota, Actinomycetota, Bacteroidota, Bacillota, and Pseudomonadota (**Figures 3A, C**). The dominant genera in these groups included Acinetobacter, Neisseria, Bacillus, Escherichia, and Prevotella (**Figures 3B, D**).

**Figure 4:**
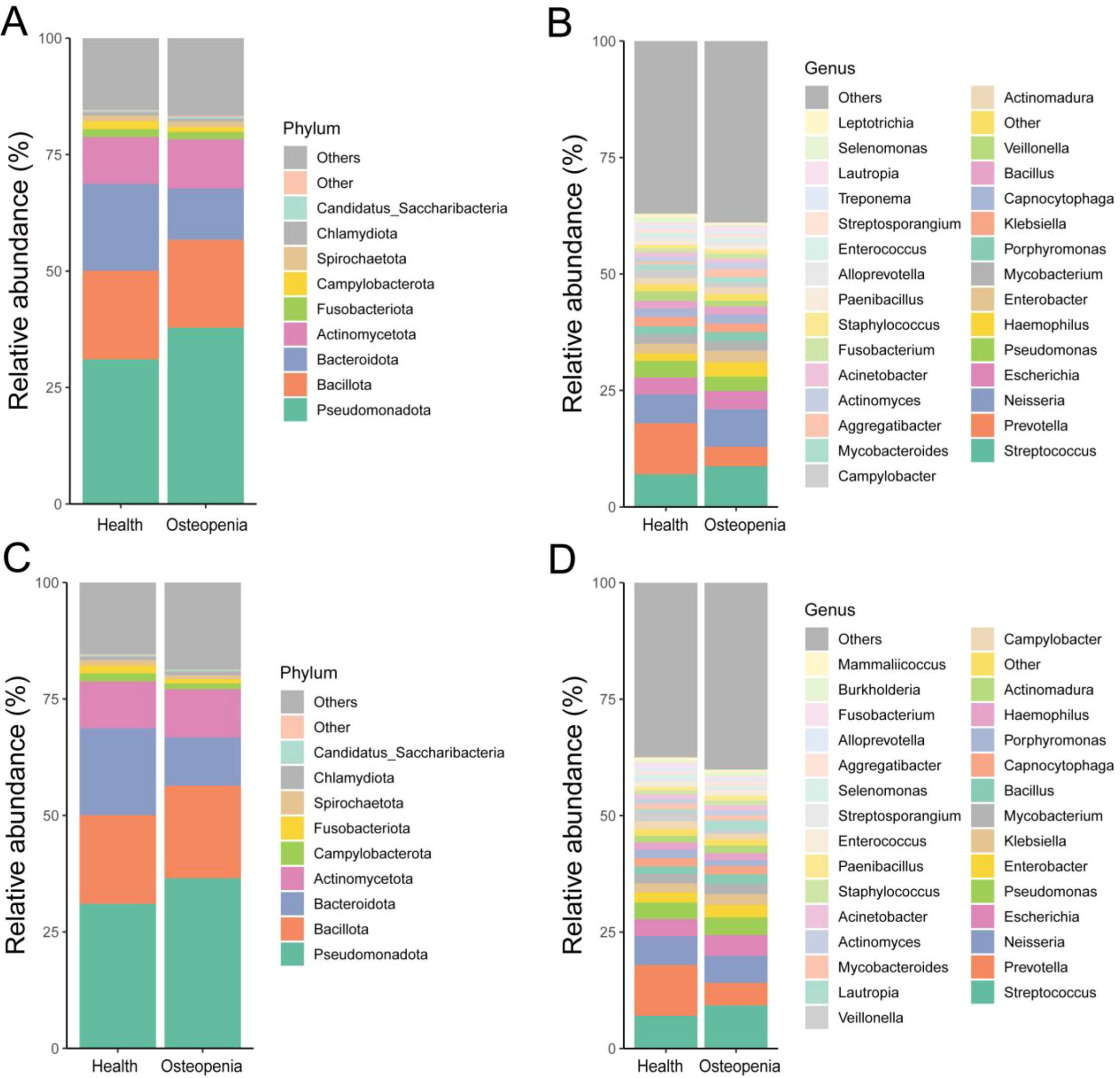
Distribution of dental plaque microbiota composition. **(A)** Distribution characteristics of microbial phyla between the Osteopenia group and the Health group. **(B)** Distribution characteristics of microbial genera between the Osteopenia group and the Health group. **(C)** Distribution characteristics of microbial phyla between the Osteoporosis group and the Health group. **(D)** Distribution characteristics of microbial genera between the Osteoporosis group and the Health group.

In dental plaque samples, the dominant phyla shared among the Health, Osteopenia, and Osteoporosis groups were Campylobacterota, Fusobacteriota, Actinomycetota, Bacteroidota, Bacillota, and Pseudomonadota (**Figures 4A, C**). The dominant genera in these groups included Pseudomonas, Escherichia, Neisseria, Prevotella, and Streptococcus (**Figures 4B, D**).

### Analysis of differential microbiota in the oral cavity

**Figure 5:**
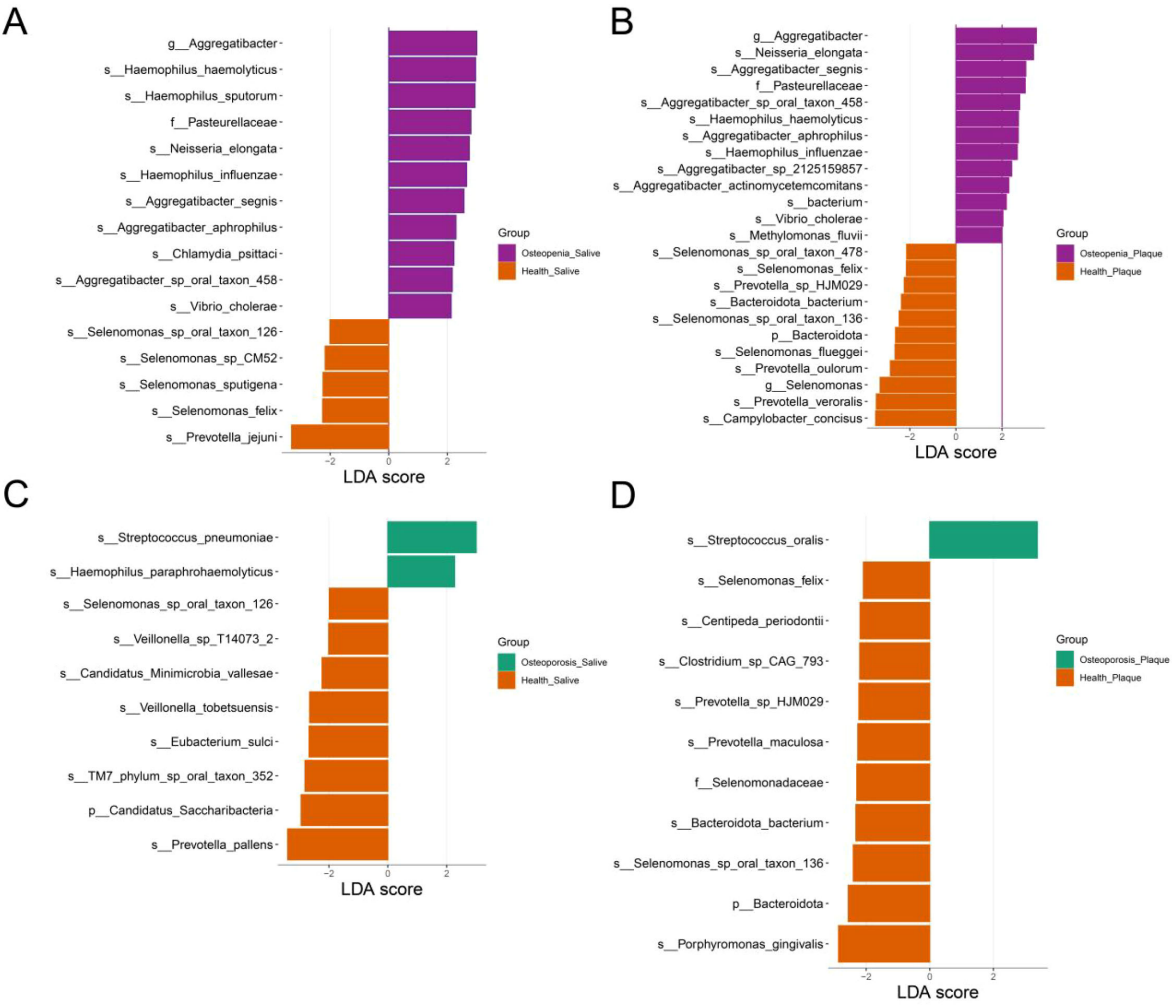
Differential oral microbiota analysis based on lEfSe method. **(A)** Sixteen differential microbes in saliva samples between the Osteopenia group and the Health group. **(B)** Twenty-four differential microbes in dental plaque samples between the Osteopenia group and the Health group. **(C)** Ten differential microbes in saliva samples between the Osteoporosis group and the Health group. **(D)** Eleven differential microbes in dental plaque samples between the Osteoporosis group and the Health group.

We used the LEfSe method to identify differentially abundant microbes, setting the LDA score threshold at 2 to pinpoint those with significant differences. Specifically, in saliva samples, compared to the Health group, the Osteopenia group exhibited higher abundance of the following microbes: Aggregatibacter, Haemophilus haemolyticus, Haemophilus sputorum, Pasteurellaceae, Neisseria elongata, Aggregatibacter segnis, Aggregatibacter aphrophilus, Chlamydia psittaci, and Vibrio cholerae (**Figure 5A**). The Osteoporosis group showed higher abundance of Streptococcus pneumoniae and Haemophilus paraphrohaemolyticus (**Figure 5C**).

In dental plaque samples, compared to the Health group, the Osteopenia group had higher abundance of Aggregatibacter, Neisseria elongata, Aggregatibacter segnis, Pasteurellaceae, Haemophilus haemolyticus, Aggregatibacter aphrophilus, Haemophilus influenzae, Aggregatibacter actinomycetemcomitans, Vibrio cholerae, and Methylomonas fluvii (**Figure 5B**). The Osteoporosis group exhibited higher abundance of Streptococcus oralis (**Figure 5D**).

### Construction of predictive models based on differential microbiota

**Figure 6:**
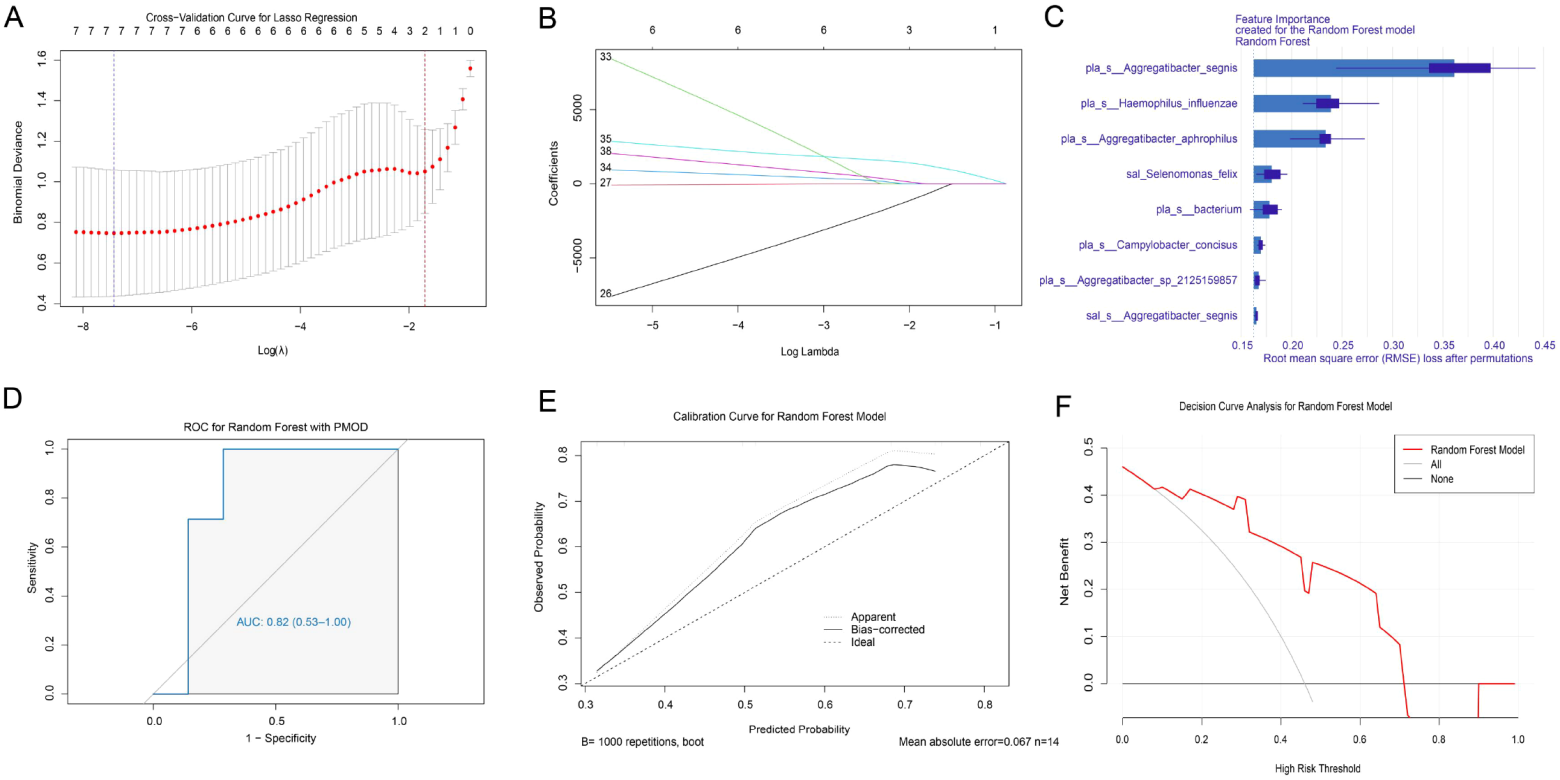
Construction and evaluation of the osteopenia vs health predictive model. **(A)** Cross-validation curve of Lasso regression. **(B)** Coefficient path plot of Lasso regression. **(C)** Feature importance plot of Lasso regression. **(D)** ROC curve of the Osteopenia vs Health predictive model. **(E)** Calibration curve of the Osteopenia vs Health predictive model. **(F)** Decision curve of the Osteopenia vs Health predictive model.

**Figure 7:**
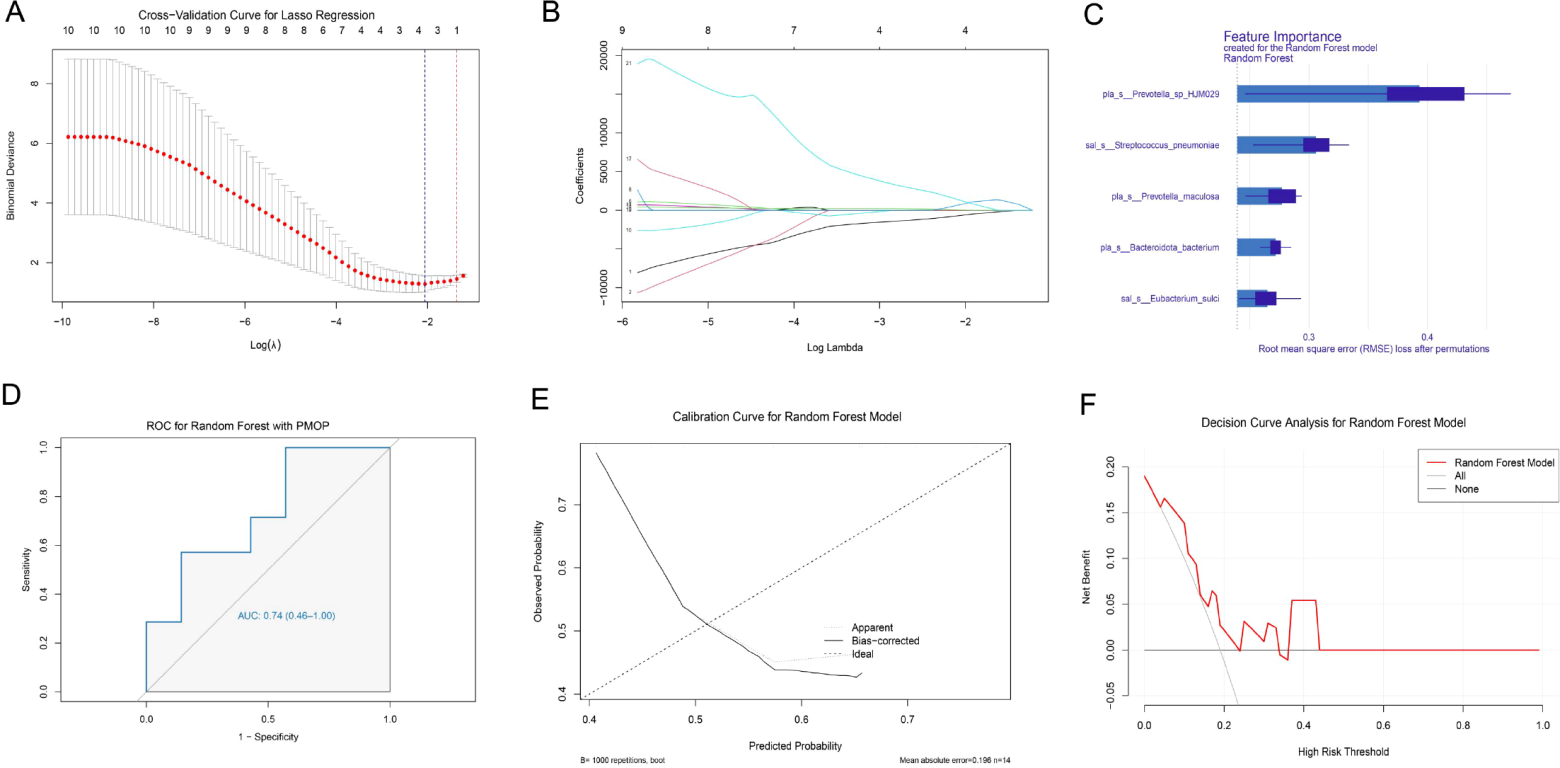
Construction and evaluation of the osteoporosis vs health predictive model. **(A)** Cross-validation curve of Lasso regression. **(B)** Coefficient path plot of Lasso regression. **(C)** Feature importance plot of Lasso regression. **(D)** ROC curve of the Osteoporosis vs Health predictive model. **(E)** Calibration curve of the Osteoporosis vs Health predictive model. **(F)** Decision curve of the Osteoporosis vs Health predictive model.

Based on the differential oral microbiota, we employed the Random Forest algorithm to identify potential biomarkers for diagnosing PMO. LASSO regression analysis was used to select features from the differential microbiota identified by LEfSe. In the Osteopenia vs. Health model, eight characteristic microbes were ultimately retained (**Figure 6C**). In the Osteoporosis vs. Health model, five characteristic microbes were retained (**Figure 7C**).

The results showed that the constructed Osteopenia vs. Health model exhibited significant predictive performance for postmenopausal bone density reduction (AUC = 0.82, 95% CI: 0.53-1.00) (**Figure 6D**). The Osteoporosis vs. Health model also demonstrated significant predictive performance for PMO (AUC = 0.74, 95% CI: 0.46-1.00) (**Figure 7D**), indicating that both models had good predictive capabilities.

The calibration curve of the Osteopenia vs. Health model closely approximated the ideal curve across most of the predicted probability range, suggesting good model calibration and consistency between predicted and observed probabilities (**Figure 6E**). In contrast, the calibration curve of the Osteoporosis vs. Health model deviated from the ideal curve in some ranges, indicating poorer calibration performance in these areas and inconsistency between predicted and observed probabilities (**Figure 7E**).

The decision curves for both the Osteopenia vs. Health model and the Osteoporosis vs. Health model showed clinical value within certain threshold ranges (**Figures 6F**, **7F**).

### Analysis of differential oral microbiota functions

The LEfSe method was used to analyze KEGG functions among the three groups, identifying significantly different functions. Subsequently, a Random Forest model was employed to rank the importance of these differential functions, and box plots were generated to illustrate the distribution differences of these functions across the groups.

**Figure 8:**
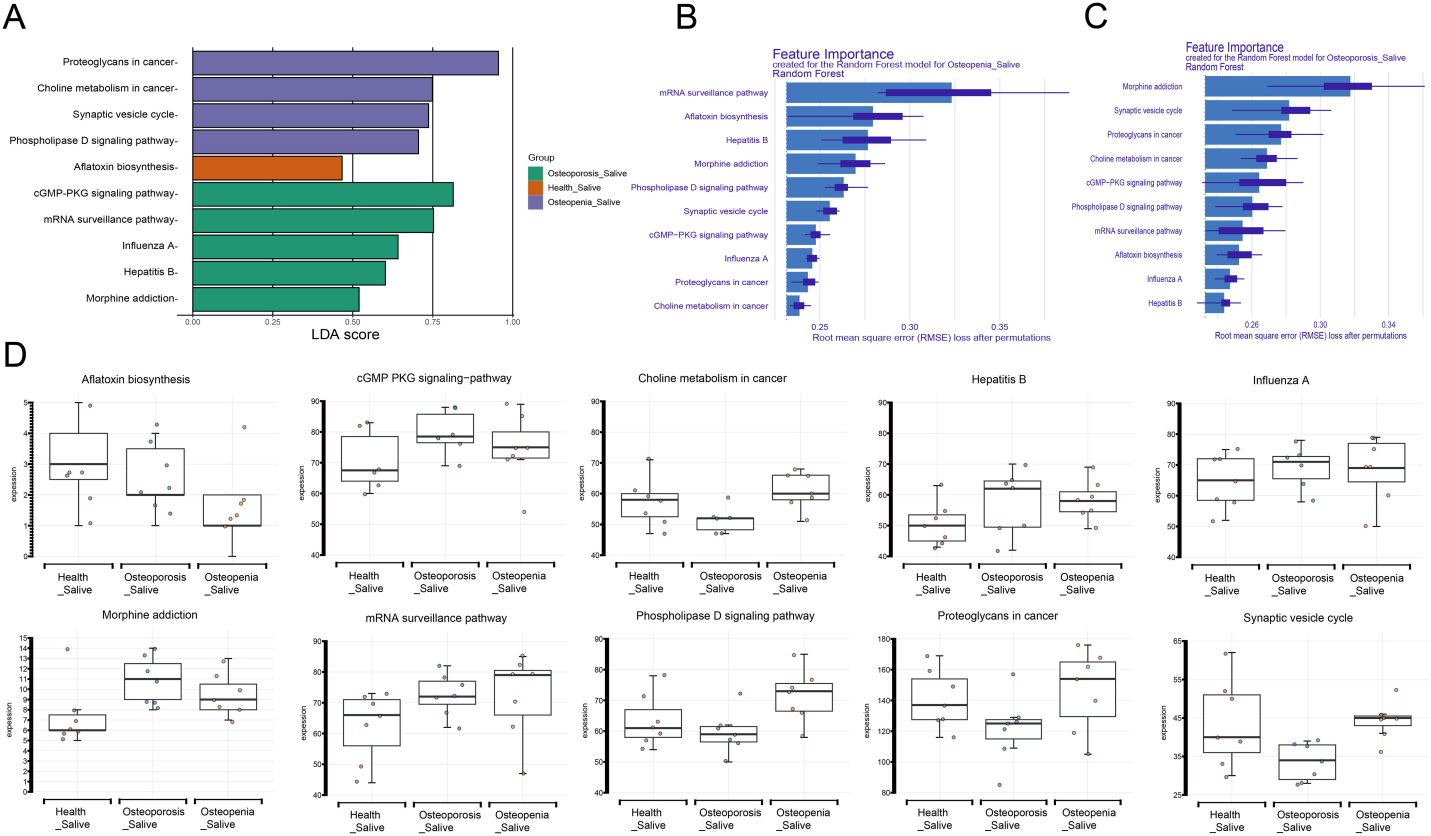
Analysis of microbial functions in saliva samples. **(A)** Differential microbial functions in saliva samples among different groups based on LEfSe analysis. **(B)** Importance ranking of microbial functions in saliva samples for the Osteopenia group. **(C)** Importance ranking of microbial functions in saliva samples for the Osteoporosis group. **(D)** Box plots showing the distribution differences of microbial functions across different groups.

In saliva samples, the abundance of the following functions was elevated in the Osteopenia group: glycan biosynthesis and metabolism in cancer, choline metabolism in cancer, synaptic vesicle cycle, and phospholipase D signaling pathway. The aflatoxin biosynthesis pathway had higher abundance in the Health group. The cGMP-PKG signaling pathway, mRNA surveillance pathway, influenza A pathway, hepatitis B pathway, and morphine addiction pathway had higher abundance in the Osteoporosis group (**Figure 8A**). Further importance ranking of these 10 functions showed that in the Osteopenia vs. Health comparison, the mRNA surveillance pathway, aflatoxin biosynthesis pathway, and hepatitis B pathway significantly contributed to distinguishing the Osteopenia group from the Health group (**Figure 8B**). In the Osteoporosis vs. Health comparison, the morphine addiction pathway, synaptic vesicle cycle pathway, and glycan biosynthesis and metabolism in cancer pathway significantly contributed to distinguishing the Osteoporosis group from the Health group (**Figure 8C**). **Figure 8D** shows the distribution differences of three microbial functions in saliva samples.

**Figure 9:**
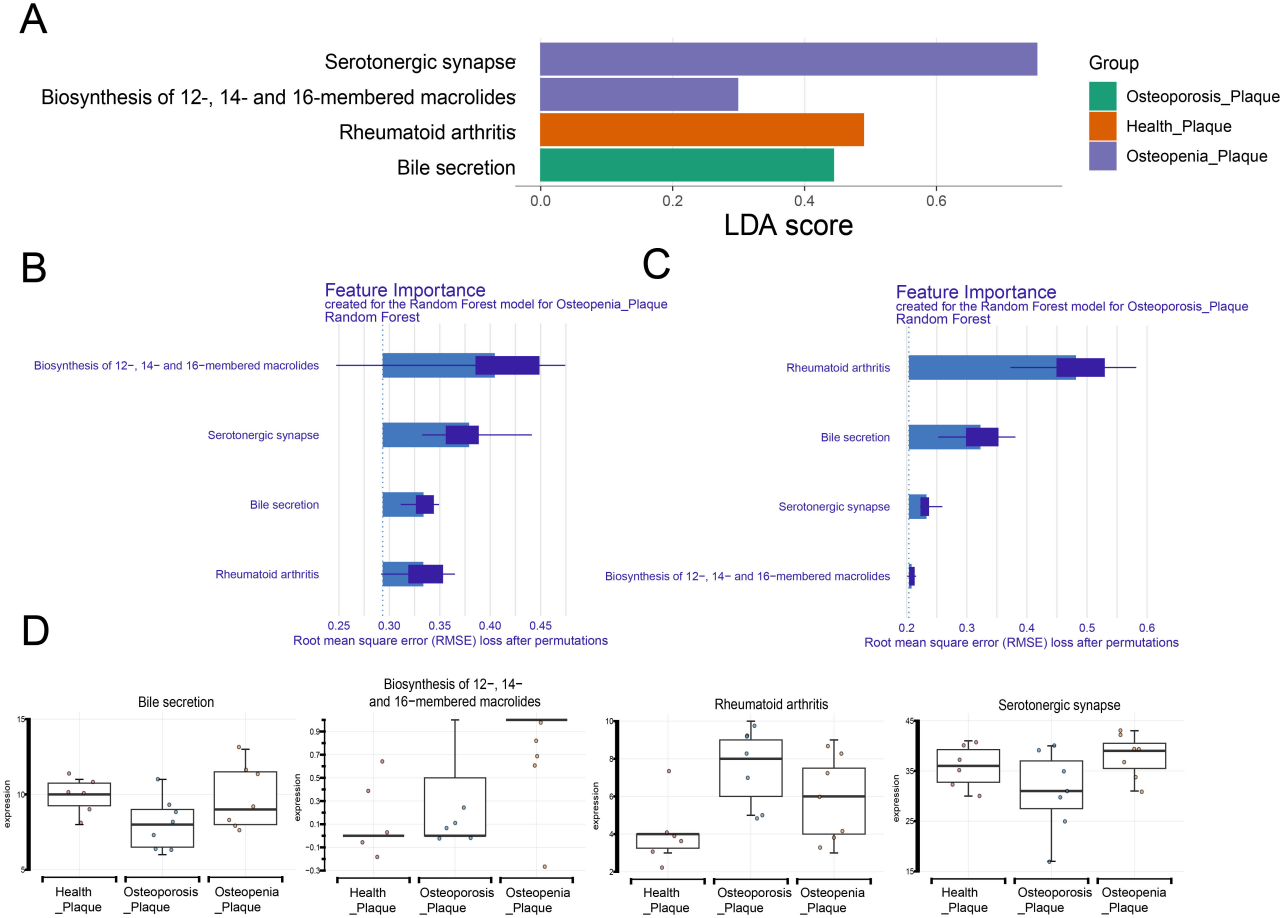
Analysis of microbial functions in dental plaque samples. **(A)** Differential microbial functions in dental plaque samples among different groups based on LEfSe analysis. **(B)** Importance ranking of microbial functions in dental plaque samples for the Osteopenia group. **(C)** Importance ranking of microbial functions in dental plaque samples for the Osteoporosis group. **(D)** Box plots showing the distribution differences of microbial functions across different groups.

In dental plaque samples, the abundance of serotonergic synapse and biosynthesis of 12-, 14-, and 16-membered macrolides was higher in the Osteopenia group. The rheumatoid arthritis pathway had higher abundance in the Health group. The bile secretion pathway had higher abundance in the Osteoporosis group (**Figure 9A**). Further importance ranking of these 4 functions showed that in the Osteopenia vs. Health comparison, the biosynthesis of 12-, 14-, and 16-membered macrolides and serotonergic synapse significantly contributed to distinguishing the Osteopenia group from the Health group (**Figure 9B**). In the Osteoporosis vs. Health comparison, the rheumatoid arthritis pathway and bile secretion pathway significantly contributed to distinguishing the Osteoporosis group from the Health group (**Figure 9C**). **Figure 9D** shows the differences in microbial functional distribution among three groups of dental plaque samples.

## Discussion

The discovery of the “gut microbiota-bone axis” has established a critical link between intestinal microbes and skeletal health, with gut microbiota profoundly influencing the balance between bone formation and resorption, thereby impacting the development of bone metabolic disorders (Indrio and Salatto, 2025). Critically, this axis plays a significant role in PMO, as evidenced by numerous studies highlighting the gut microbiota’s contribution to bone homeostasis dysregulation in this population (Fuhrman et al., 2014; Nilsson et al., 2018; Tu et al., 2020). A growing number of studies have identified interactions between the oral microbiota and the gut microbiota, leading to the concept of the “oral-gut axis”. Studies confirm significant correlations between microbial compositions in these sites in healthy individuals, suggesting potential migration of oral microbes to the gut via swallowed saliva or hematogenous spread during oral inflammation, influencing gut ecology (Ding and Schloss, 2014; Atarashi et al., 2017; Bao et al., 2022). Furthermore, oral dysbiosis, characterized by an increase in opportunistic pathogens like Streptococcus, Actinomyces, Veillonella, and Fusobacterium, has been associated with bone conditions such as osteoarthritis, pointing towards a broader oral-gut-bone axis (Chen et al., 2018). However, despite this emerging paradigm of an “oral-gut-bone axis,” direct investigations into the specific relationships between the oral microbiota and BMD changes in postmenopausal women remain scarce. To address this crucial knowledge gap, our study employs metagenomic sequencing to perform a comprehensive oral microbiome analysis on postmenopausal women categorized by BMD. This approach is anticipated to yield new understanding into the potential impact of the oral microbiome on PMO pathophysiology through the oral-gut-bone axis.

In this study, we employed metagenomic sequencing to analyze the relationship between oral microbiota and PMO. Although no significant differences were observed in alpha diversity among groups, PCoA analysis revealed significant beta diversity variations in salivary microbiota between the Osteoporosis and Health groups, suggesting that specific microbial communities may be associated with different PMO statuses.

Our analysis revealed a significant increase in multiple potential opportunistic oral pathogens in the Osteopenia group compared to the Health group across both saliva and dental plaque samples, including Aggregatibacter, Haemophilus haemolyticus, Haemophilus sputorum, Pasteurellaceae, Neisseria elongata, Aggregatibacter segnis, and Aggregatibacter aphrophilus. Notably, most of these microorganisms belong to the Proteobacteria phylum, a finding consistent with previous studies (He et al., 2020; Palacios-González et al., 2020; Zhao et al., 2021). These results suggest an association between increased relative abundance of Proteobacteria and reduced bone mineral density. This correlation may stem from the well-established link between Proteobacteria-associated dysbiosis and inflammation, which is known to promote osteoclast activation and potentially adversely affect bone health (Steves et al., 2016). Previous research has specifically demonstrated that elevated Pasteurellaceae abundance correlates with increased osteoporosis risk (Zeng et al., 2024). In the LEfSe analysis, Aggregatibacter exhibited the highest score in the Osteopenia group, suggesting its most significant association with reduced bone mineral density. Notably, Aggregatibacter actinomycetemcomitans within this genus has been demonstrated to translocate from periodontal tissues into systemic circulation, promoting systemic inflammatory responses that subsequently lead to decreased whole-body bone mineral density (Zhang et al., 2020b). Conversely, our findings revealed higher abundances of Streptococcus pneumoniae and Streptococcus oralis in the Osteoporosis group compared to the Health group. As one of the primary pathogenic bacteria in periodontitis, Streptococcus contribute to localized pH reduction through biofilm formation and metabolic byproducts, thereby activating host immune responses and exacerbating gingival inflammation and alveolar bone resorption (Ji et al., 2015). In patients with periodontitis, pro-inflammatory factors disseminate systemically through circulation, activating osteoclast differentiation and resulting in systemic bone loss that ultimately promotes osteoporosis development (Yu and Wang, 2022). The study by Peng demonstrated a significant association between osteoporosis and periodontitis, particularly pronounced in postmenopausal women with severe periodontitis (Peng et al., 2023), which further supports the potential impact of Streptococcus on bone health.

Based on oral microbiome characteristics, we successfully constructed predictive models for postmenopausal bone density reduction (Osteopenia vs Health) and osteoporosis (Osteoporosis vs Health) using random forest algorithm combined with LASSO regression analysis, providing a novel microbial biomarker strategy for the early non-invasive diagnosis of PMO. Both models demonstrated good discriminative ability (AUC values of 0.82 and 0.74, respectively), with the notably higher AUC value of the Osteopenia model suggesting that oral microbiome may exhibit sensitivity to bone mass changes even before the clinical onset of osteoporosis. Notably, the Osteopenia model showed superior calibration performance, with predicted probabilities closely matching actual observed probabilities, while the calibration deviation observed in the Osteoporosis model might stem from either increased complexity of host-microbiome interactions at the osteoporosis stage or sample size limitations - a finding that warrants validation through expanded cohort studies. From a clinical translation perspective, decision curve analysis confirmed that both models offer clear net clinical benefits within specific threshold ranges, demonstrating particular potential for screening high-risk populations. This study innovatively utilizes oral microbiome as biomarker source, offering easier implementation compared to traditional invasive detection methods. However, it’s important to note that microbiome data can be influenced by factors such as diet and oral hygiene practices.

We employed LEfSe analysis to examine differential KEGG functional pathways among the three study groups. In saliva samples, the Osteopenia group showed elevated abundance in proteoglycans in cancer, choline metabolism in cancer, synaptic vesicle cycle, and phospholipase D signaling pathways, while the Osteoporosis group exhibited increased abundance in cyclic GMP-protein kinase G (cGMP-PKG) signaling pathway, mRNA surveillance pathway, influenza A pathway, hepatitis B pathway, and morphine addiction pathway. In dental plaque samples, the Osteopenia group demonstrated higher abundance in serotonergic synapse and biosynthesis of 12-, 14-, and 16-membered macrolides pathways. Certain oral bacteria can translocate to the gut through swallowing, influencing gut microbiota composition and function. Gut microbial metabolism of choline, betaine, and carnitine produces trimethylamine N-oxide (TMAO). Li et al. demonstrated that reduced TMAO levels may attenuate bone loss in elderly mice with low bone mass by alleviating inflammatory responses (Li et al., 2019). Another study found elevated serum TMAO levels in postmenopausal women with hip fractures were associated with increased fracture risk, suggesting TMAO may promote osteoporosis and fracture occurrence in postmenopausal women (Liu et al., 2020). Zhao et al. further confirmed that TMAO promotes osteoclast differentiation through NF-κB signaling pathway activation (Zhao et al., 2024), while excessive osteoclast activation accelerates bone loss and significantly increases osteoporosis and osteoarthritis risk (Veis and O’Brien, 2023). It is worth mentioning that we also found that the abundance of the hepatitis B pathway increased in the osteoporosis group. Previous studies have found that patients with chronic hepatitis B, especially those with liver cirrhosis, have significantly lower bone mineral density than healthy people, and the incidence of osteoporosis increases with the severity of liver cirrhosis (Zhang et al., 2020a). Hepatitis B virus infection induces inflammatory cytokine secretion (e.g., TNF-α, IL-1β, IL-6), which promotes bone resorption and reduces bone formation by activating osteoclasts and inhibiting osteoblast function (Byrne et al., 2014). Additionally, impaired liver function in cirrhosis patients may cause vitamin D metabolic abnormalities, further exacerbating osteoporosis risk (Konstantakis et al., 2016). Gut-derived serotonin binds to Htr1B receptors on osteoblasts, inhibiting their differentiation, proliferation, and mineralization, thereby suppressing bone formation and increasing osteoporosis risk (Duffuler et al., 2024). These findings collectively suggest that oral microbiota may influence gut microbiota composition and function through a “swallowing-translocation” mechanism. Microbial metabolites (e.g., TMAO) and inflammatory cytokines act through multiple pathways to form a complex “oral-gut-bone axis” regulatory network, ultimately leading to the dual imbalance of reduced bone formation and increased bone resorption.

However, several limitations of this current preliminary study should be acknowledged. First, the exploratory nature of this research and its findings must be interpreted in the context of a modest sample size (n=21). While the significant p-values from PERMDISP analyses (P = 0.036; P = 0.041) suggest differences in microbial communities across PMO subjects with varying bone mineral density, these results warrant caution due to potential sensitivity to the limited sample. The absence of multiple test correction also implies that the identified microbial markers should be considered hypothesis-generating. Therefore, the primary validation of our findings awaits future large-scale epidemiological studies. Second, although we observed correlations between oral microbial communities, functional pathways, and PMO, the cross-sectional design precludes causal inference. The specific mechanisms linking the oral microbiota to bone homeostasis remain uninvestigated and represent a critical area for future mechanistic inquiry. For instance, germ-free animal models or fecal microbiota transplantation experiments could help elucidate the impact of specific oral microbes on bone metabolism, offering more direct evidence for an oral-gut-bone axis. Finally, as LEfSe was used for feature prescreening prior to LASSO/random forest modeling, the reported AUC may be susceptible to overfitting and should be interpreted as an exploratory indicator of discriminative potential rather than as a validated diagnostic measure.

Despite these limitations, this study serves as an initial exploration of the relationship between the oral microbiome and bone homeostasis in postmenopausal women with osteoporosis. Our metagenomic analyses reveal meaningful associations between oral microbial composition, functional pathways, and bone density, supporting the relevance of the oral microbiome in PMO. These findings provide a new direction and rationale for further investigation into how oral microbes may influence systemic bone health. We hope this work encourages more researchers to examine the role of oral microbiota in PMO bone regulation. Subsequent studies should include larger clinical cohorts to identify more reliable biomarkers for PMO screening, as well as experimental validations—using targeted bacterial strains or mechanistic models—to clarify the underlying pathways through which the oral microenvironment influences systemic bone density.

## Conclusions

In this exploratory study, we employed metagenomic sequencing to investigate the relationship between oral microbiota and PMO. Our analysis revealed compositional and functional alterations in the oral microbiota that were associated with BMD status. Notably, taxa such as Aggregatibacterand Haemophilus haemolyticuswere observed to be more abundant in groups with osteopenia and osteoporosis. A predictive model built upon these differential microbial features showed promise for future research, meriting further validation. Functional profiling suggested potential associations with inflammatory and metabolic pathways. Collectively, these findings contribute preliminary evidence supporting an exploratory link between the oral microbiota and PMO, suggesting that microbial profiles may hold value as a candidate for non-invasive risk assessment. Future prospective studies with larger cohorts are warranted to confirm these associations and elucidate any causal mechanisms.

## Data Availability

The raw metagenomic sequencing data generated in this study have been deposited into a public repository. The data have been uploaded to the National Center for Biotechnology Information Sequence Read Archive and are accessible under the BioProject accession number PRJNA1345159.All other data are available upon request from the authors.
