# Supplementary material for: Correlation between oral microbial characteristics and overall bone density of Postmenopausal women based on macrogenomic analysis
Source: Front Cell Infect Microbiol. 2025 Dec 1;15:1663645. doi: 10.3389/fcimb.2025.1663645 (PMC12702920; doi:10.3389/fcimb.2025.1663645)
Supplement: Supplementary file 1 [file DataSheet1.pdf]

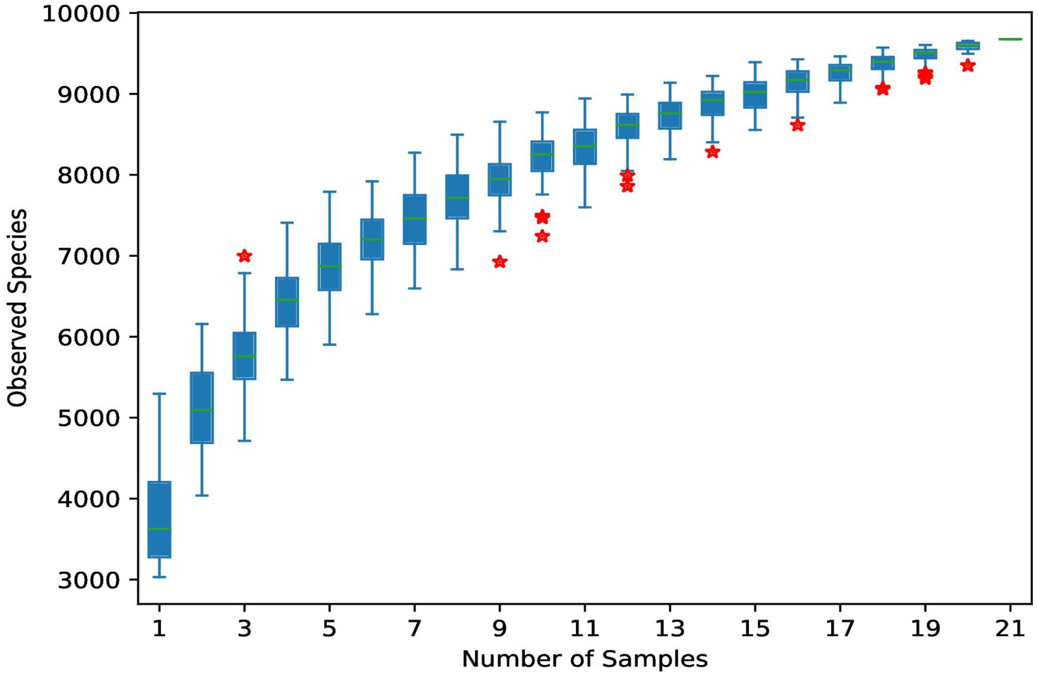

Supplementary Figure 1. Species-based cumulative box plot.

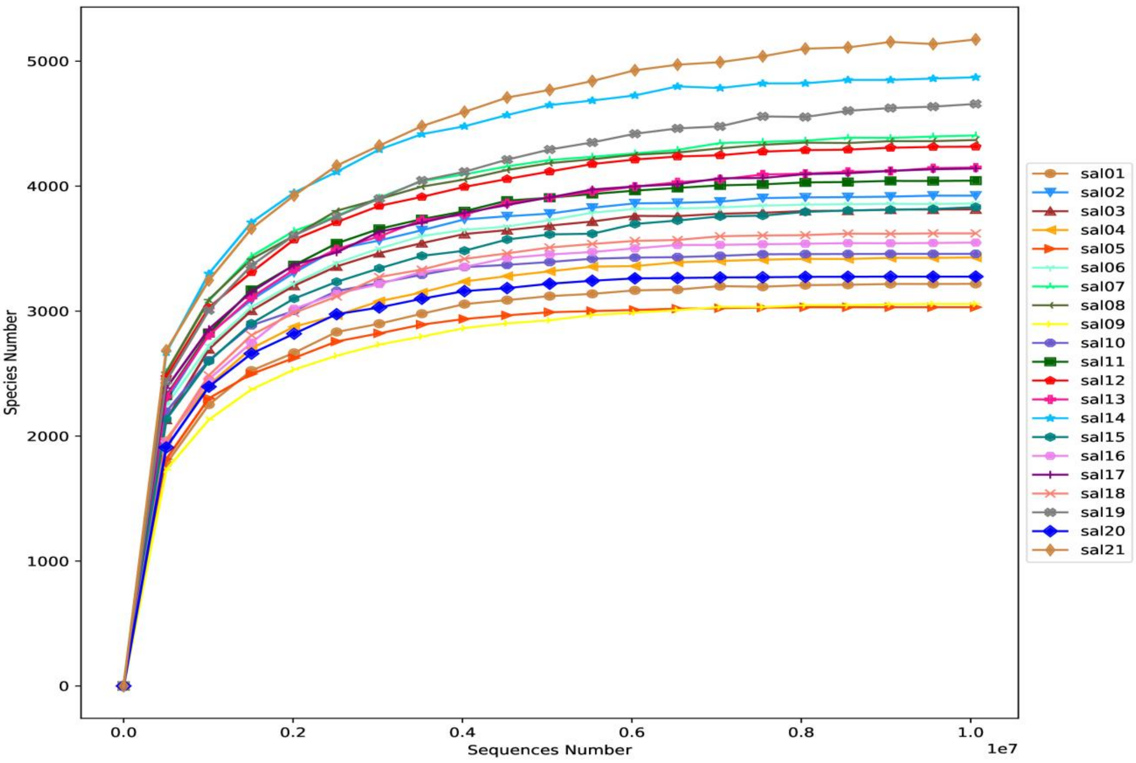

2 **Supplementary Figure 2.** Dilution curve based on the number of species sequences per sample.

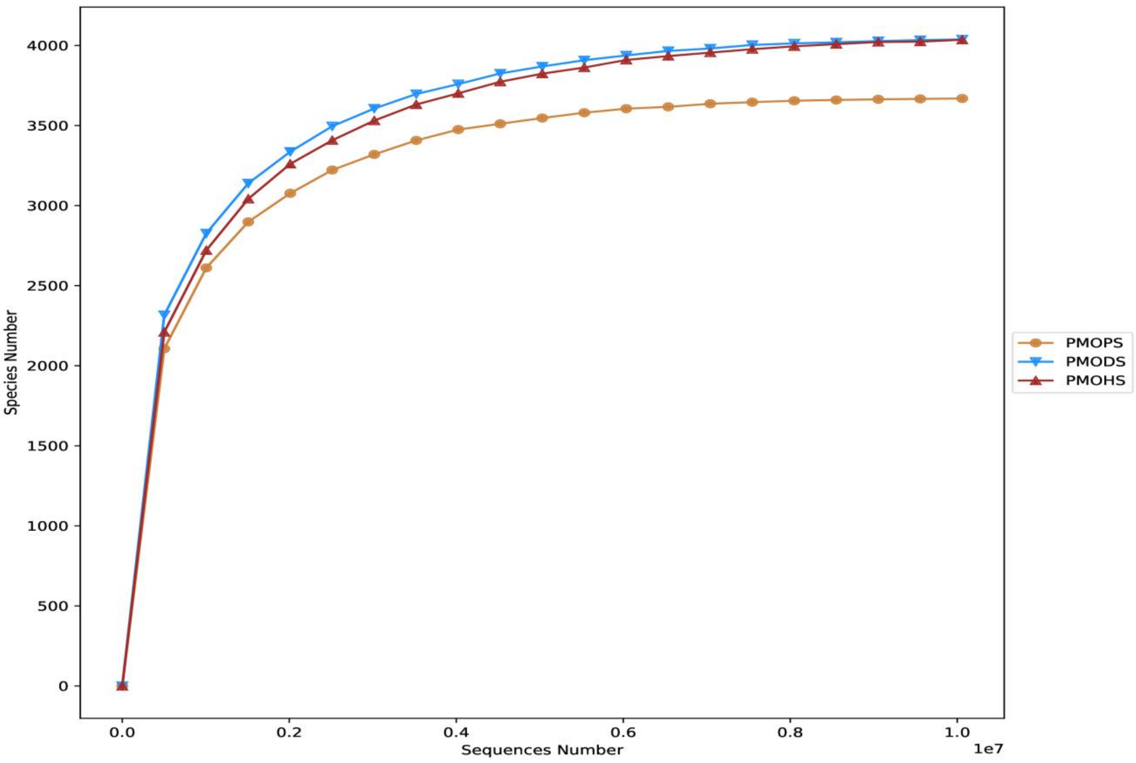

Supplementary Figure 3. Dilution curve based on the number of species sequences per group.

Supplementary Table 1. Sample Coverage Table.

sample	Goods_coverage
sal01	1
sal02	1
sal03	1
sal04	1
sal05	1
sal06	1
sal07	1
sal08	1
sal09	1
sal10	1
sal11	1
sal12	1
sal13	1
sal14	1
sal15	1
sal16	1
sal17	1
sal18	1
sal19	1
sal20	1
sal21	1

Supplementary Table 2. PERMANOVA Table for Beta Diversity Adjusted for BMI and Year.

Matrix	Comparison	Metric	R2	F	P	Permutations	n
Saliva	Health vs Osteoporosis	PERMANOVA (Bray) _group	0.20095	2.7769	0.0914	9999	14
Saliva	Health vs Osteoporosis	PERMANOVA (Jaccard) _group	0.10221	1.3512	0.0607	9999	14
Saliva	Health vs Osteoporosis	PERMANOVA (Bray) _BMI	0.00945	0.1306	0.9504	9999	14
Saliva	Health vs Osteoporosis	PERMANOVA (Bray) _year	0.10723	1.4818	0.2263	9999	14
Saliva	Health vs Osteoporosis	BETADISPER (Bray) _permutest		6.2013	0.0303	9999	14
Saliva	Health vs Osteoporosis	partial dbRDA (Bray) _overall		2.7725	0.0946	9999	14
Saliva	Health vs Osteoporosis	partial dbRDA (Bray) _group		2.7725	0.0917	9999	14
Saliva	Health vs Osteopenia	PERMANOVA (Bray) _group	0.07525	1.0686	0.3448	9999	14
Saliva	Health vs Osteopenia	PERMANOVA (Jaccard) _group	0.07567	0.97	0.4586	9999	14
Saliva	Health vs Osteopenia	PERMANOVA (Bray) _BMI	0.01544	0.2192	0.8734	9999	14
Saliva	Health vs Osteopenia	PERMANOVA (Bray) _year	0.16761	2.3801	0.1255	9999	14
Saliva	Health vs Osteopenia	BETADISPER (Bray) _permutest		6.9497	0.0212	9999	14
Saliva	Health vs Osteopenia	partial dbRDA (Bray) _overall		1.0665	0.3499	9999	14
Saliva	Health vs Osteopenia	partial dbRDA (Bray) _group		1.0665	0.3572	9999	14
Plaque	Health vs Osteoporosis	PERMANOVA (Bray) _group	0.13278	1.6798	0.176	9999	14
Plaque	Health vs Osteoporosis	PERMANOVA (Jaccard) _group	0.08291	1.0734	0.2566	9999	14
Plaque	Health vs Osteoporosis	PERMANOVA (Bray) _BMI	0.02064	0.2611	0.9251	9999	14
Plaque	Health vs Osteoporosis	PERMANOVA (Bray) _year	0.08044	1.0176	0.3651	9999	14
Plaque	Health vs Osteoporosis	BETADISPER (Bray) _permutest		2.5068	0.1418	9999	14
Plaque	Health vs Osteoporosis	partial dbRDA (Bray) _overall		1.6746	0.1797	9999	14
Plaque	Health vs Osteoporosis	partial dbRDA (Bray) _group		1.6746	0.18	9999	14
Plaque	Health vs Osteopenia	PERMANOVA (Bray) _group	0.07302	0.9558	0.4074	9999	14
Plaque	Health vs Osteopenia	PERMANOVA (Jaccard) _group	0.06915	0.8616	0.8367	9999	14
Plaque	Health vs Osteopenia	PERMANOVA (Bray) _BMI	0.0152	0.1989	0.9878	9999	14
Plaque	Health vs Osteopenia	PERMANOVA (Bray) _year	0.12557	1.6436	0.1849	9999	14
Plaque	Health vs Osteopenia	BETADISPER (Bray) _permutest		3.5572	0.0897	9999	14
Plaque	Health vs Osteopenia	partial dbRDA (Bray) _overall		0.9551	0.4002	9999	14
Plaque	Health vs Osteopenia	partial dbRDA (Bray) _group		0.9551	0.4142	9999	14

Supplementary Text1. The consent form template.

Informed Consent

Project Title: Investigating the Mechanism of PMOP Oral Microbiome Metagenomic Analysis on Bone Metabolism

Research institute: Shenzhen Maternal and Child Health Hospital

Principal investigators (responsible physicians): Wu Min, Liu Man, Huang Xixi, Ye Chanjuan

You will be invited to participate in a clinical study. This informed consent form provides you with information to help you decide whether to participate in this clinical study. Please read it carefully and ask the investigator in charge of the study for any questions.

Your participation in this study is voluntary. This study has been reviewed and approved by the Institutional Ethics Committee.

Research Objective: Postmenopausal osteoporosis is emerging as a major public health concern in developing countries, with its complications severely impacting the quality of life for postmenopausal and elderly women while causing substantial economic losses. Early detection and proactive prevention of postmenopausal osteoporosis are crucial for improving womens health outcomes. Building on previous research and guided by the fundamental theories of the "gut microbiota-bone axis" and the oral-gut microbiota relationship, this study investigates the effects of oral microbiota on whole-body bone density in postmenopausal women. The research aims to identify early biomarkers for postmenopausal osteoporosis and develop novel targeted therapeutic strategies for clinical prevention and treatment.

Research process: The subjects were required to cooperate with the doctor for relevant questionnaire survey, oral examination, oral saliva collection and plaque collection.

Risks and Discomfort: The digital panoramic X-ray and bone density measurement devices emit minimal radiation, posing no health risks. For saliva collection, participants must avoid brushing teeth on the morning of the procedure and the night before, refrain from eating for at least two hours prior to sampling, and provide non-irritating saliva.

Benefit: Postmenopausal women can gain early insights into their oral health and overall bone density, enabling timely prevention and treatment. Participants who submit saliva and plaque samples will receive personalized oral health guidance and a Swedish TEPE toothbrush set.

Cost: The research costs required for this project will be borne by the research group, and you do not need to bear any costs required for the research process itself.

As a research subject, you have the following responsibilities: Provide truthful information about your medical history and current physical condition; Inform the research doctor of any discomfort you experience during the study; Do not take restricted medications, foods, etc.; Inform the research doctor if you have recently participated in or are currently participating in other studies.

Privacy Policy: If you choose to participate in this study, your involvement and personal data will remain strictly confidential. Your tissue samples will be labeled with a research code rather than your name. Any identifying information will not be shared with third parties unless you consent. All participants and the research sponsor are required to maintain your anonymity. Your records will be securely stored in a locked archive cabinet accessible only to authorized researchers. Government regulators or ethics review committee members may review your personal data at the research facility when necessary to ensure compliance. When the study findings are published, no personal information will be disclosed.

You may choose not to participate in this study or withdraw from the study at any time by notifying the investigator. Your data will not be included in the study results and your medical benefits and rights will not be affected.

If you need additional treatment, or if you do not comply with the study protocol, or if you have a study-related injury or for any other reason, the study physician may discontinue your participation in the study.

You can keep abreast of information and progress related to this study at any time. If you have any questions about this study, or if you experience any discomfort or injury during the study, or if you have any questions about the rights of participants in this study, you can contact the investigator by phone.

********* ↓ Informed Consent Form Signature Page ↓ ***********

Informed Consent Form Signature Page

Informed consent statement:

I have carefully read the entire informed consent form, the researchers have explained the study to me, and I have had the opportunity to discuss the study with the researchers and ask questions, and all the questions I have asked have been answered satisfactorily.

I am aware of the risks and benefits of participating in this study, and I have decided to participate in this study on a voluntary basis. I have the right to withdraw from this study at any time.

The records of this study and my medical records may be viewed by authorized representatives of the investigators, the regulatory agency, or the ethics committee related to my participation in this study. I allow these people to access my medical records and I know that this information will be kept confidential.

I agree to participate in this study.

Signature of the subject (or legal representative) :___________________________

Contact number: _____________________Date: ______________________________

(Note: If the subject is not competent, the signature of an agent is required.)

The researchers stated:

I will strictly comply with the laws and regulations related to clinical research and relevant behavioral preparations, ensure that the medical treatment and legal rights of the subjects are fully respected, and strictly protect the privacy of the subjects.

Signature of the researcher:___________________________

Contact number: _____________________Date: ______________________________
